# Nanodelivery System for Ovsynch Protocol Improves Ovarian Response, Ovarian Blood Flow Doppler Velocities, and Hormonal Profile of Goats

**DOI:** 10.3390/ani12111442

**Published:** 2022-06-03

**Authors:** Nesrein M. Hashem, Hossam R. EL-Sherbiny, Mohamed Fathi, Elshymaa A. Abdelnaby

**Affiliations:** 1 Department of Animal and Fish Production, Faculty of Agriculture (El-Shatby), Alexandria University, Alexandria 21545, Egypt; 2 Theriogenology Department, Faculty of Veterinary Medicine, Cairo University, Giza 12211, Egypt; hossamelsherbiny7353575@cu.edu.eg (H.R.E.-S.); mido2022@yahoo.com (M.F.); elshymaa.ahmed@cu.edu.eg (E.A.A.)

**Keywords:** nanodelivery system, GPG, estrous synchronization, blood flow, ovary

## Abstract

**Simple Summary:**

Drug delivery systems depending on nanotechnology have been recently created to improve biological activity of drugs including hormones. Nanoformulated drugs have different pharmacokinetic properties in biological systems compared to their conventional forms due to the acquired new physicochemical properties such as lesser size, high size-to-weight ratio, and different surface charges and shapes. These properties enable them to efficiently deliver into target sites coping with existing biological barriers. Ovsynch protocol, GPG, is one of the most important estrous synchronization protocols due to the possibility of applying timed insemination with this protocol. This protocol is commonly used in many farm animals, as it aids in herd management and eliminates the need for the detection of estrus. However, there are still some shortcomings that restrict the outcomes of this protocol, such as scattering ovulation time, short luteal phase and inadequate luteal function, and low conception rates. Therefore, this study aims to evaluate ovarian response, blood flow of the ovarian and luteal arteries, and hormonal profile of goats receiving either a standard Ovsynch protocol or an Ovsynch protocol delivered via a nanodelivery system with different dosages of hormones. The present study is the first one that poses the idea that the nanodelivery system for the Ovsynch protocol enables lower hormone dose administration and improves the results of the protocol by inducing tighter synchrony of ovulation and better luteal function of synchronized goats.

**Abstract:**

Fifteen cyclic, multiparous goats were equally stratified and received the common Ovsynch protocol (GPG: intramuscular, IM, injection of 50 mg gonadorelin, followed by an IM injection of 125 µg cloprostenol 7 days later, and a further IM injection of 50 mg gonadorelin 2 days later) or the Ovsynch protocol using nanofabricated hormones with the same dosages (NGPG) or half dosages (HNGPG) of each hormone. The ovarian structures and ovarian and luteal artery hemodynamic indices after each injection of the Ovsynch protocol using B-mode, color, and spectral Doppler scanning were monitored. Levels of blood serum progesterone (P_4_), estradiol (E_2_), and nitric oxide (NO) were determined. After the first gonadotrophin-releasing hormone (GnRH) injection, the number of large follicles decreased (*p* = 0.02) in NGPG and HNGPG, compared with GPG. HNGPG resulted in larger corpus luteum (CL) diameters (*p* = 0.001), and improved ovarian and luteal blood flow, compared with GPG and NGPG. Both NGPG and HNGPG significantly increased E_2_ and NO levels compared with GPG. HNGPG increased (*p* < 0.001) P_4_ levels compared with GPG, whereas NGPG resulted in an intermediate value. After prostaglandin F_2α_ (PGF_2α_) injection, HNGPG had the largest diameter of CLs (*p* = 0.001) and significantly improved ovarian blood flow compared with GPG and NGPG. Both NGPG and HNGPG increased (*p* = 0.007) NO levels, compared with GPG. E_2_ level was increased (*p* = 0.028) in HNGPG, compared with GPG, whereas NGPG resulted in an intermediate value. During the follicular phase, HNGPG increased (*p* = 0.043) the number of medium follicles, shortened (*p* = 0.04) the interval to ovulation, and increased (*p* < 0.001) ovarian artery blood flow and levels (*p* < 0.001) of blood serum P_4_, E_2_, and NO, compared with GPG and NGPG. During the luteal phase, the numbers of CLs were similar among different experimental groups, whereas the diameter of CLs, luteal blood flow, and levels of blood serum P_4_ and NO increased (*p* < 0.001) in HNGPG, compared with GPG and NGPG. Conclusively, the nanodelivery system for the Ovsynch protocol could be recommended as a new strategy for improving estrous synchronization outcomes of goats while enabling lower hormone dose administration.

## 1. Introduction

One of the most widely used assisted reproductive techniques in farm animals is estrous cycle synchronization. Several estrous synchronization protocols based on the use of P_4_ or PGF_2α_ analogs as key regulatory hormones have been developed [1]. Nevertheless, these protocols result in sufficient synchrony of the estrous cycle, and they do not eliminate the need for estrus detection. With the widespread of the artificial insemination process and the increased intensive livestock production system, the need for more practicable estrous synchronization protocols has become crucial [2]. The inclusion of gonadotropins, specifically GnRH, in estrous synchronization procedures, allows for more regulation of ovarian function by modulating follicular waves and the corpus luteum life span [3,4]. The full control of the ovarian activity helps set the ovulation time and thus apply fixed time-artificial insemination, eliminating the need for estrous detection as one of the most laborious farm works. To date, the popular estrous synchronization protocol that confers these advantages is “Ovsynch” or “GPG,” which includes a GnRH-PGF_2α_-GnRH treatment sequence [5,6]. This protocol has gained increasing attention, and it is applied to synchronize estrous/ovarian cycles in different farm animal species, including goats. In goats, this procedure aids in herd management, particularly large herds, by concentrating on kidding and related farm practices in a short time [4]. Nevertheless, promising synchronization results have been obtained when applying such a protocol in various farm animals, including dairy cows, sheep, and goats [3,6,7]. There are still some defects that restrict the outcomes of this protocol, such as scattering ovulation time, short luteal phase and inadequate luteal function, and low conception rates [3,8]. Part of estrous synchronization protocols efficiency is ascribed to the pharmacokinetics of hormones and their bioavailability [1]. Because both GnRH and PGF_2α_ have a small molecular weight and short half-life, improving the pharmacokinetics and bioavailability of these hormones may enhance their biological action and estrous synchronization outcomes [9]. Recently, nanotechnology and drug delivery systems have been used to improve drugs, hormones, and biological activity. Nanodelivered drugs have varying pharmacokinetic properties in biological systems different from their conventional forms [10]. Nanodrugs have lesser size, high size to weight ratio, and different surface charge and shapes, compared with the original drugs [11]. These criteria can be controlled by applying different carrying materials and fabrication conditions, aiming for better delivery of drugs into target sites coping with different biological barriers (e.g., lytic enzymes and blood barriers). Few studies have referred to the improved biological activity of nanofabricated GnRH compared with the conventional form. For example, nano-GnRH improved artificial insemination outcomes of rabbits [12] and luteal function of pregnant goats [9] even when GnRH dosage was reduced. Hence, this study aimed to evaluate ovarian response, Doppler sonographic analysis of ovarian and luteal arteries, and hormonal profile of goats synchronized for estrus with conventional Ovsynch protocol or nanodelivery Ovsynch protocol.

## 2. Materials and Methods

### 2.1. Fabrication and Characterization of Hormone-Conjugated Nanoparticles

Chitosan (Cat No. AL1234 00100; Alpha Chemica, Maharashtra, India) and sodium tripolyphosphate (TPP; Thermo Fisher GmbH, Kandel, Germany) were used to create a nanocarrier polymer using the ionic gelation method described by Hashem et al. [9]. Briefly, chitosan (0.1%, *w*/*v*) was vigorously stirred in an aqueous acidic solution (1%, *v*/*v*) to obtain chitosan cation nanoparticles. Furthermore, we prepared an aqueous solution of TPP (0.1 %, *w*/*v*). The chitosan-TPP nanoparticles complex were prepared by slowly dropping the TPP solution into the chitosan solution (2 chitosan: 1 TPP) under constant magnetic stirring (1200 rpm) for 2 h. Either gonadorelin solution (Ovurelin, 100 µg gonadorelin (as acetate), /mL, Bayer New Zealand Limited, Manukau, Auckland, New Zealand) or prostaglandin F_2α_ solution (PGF_2α_, Estrumate, 250 µg cloprostenol/mL, equivalent to 263 µg cloprostenol sodium/mL, Intervet/Merck Animal Health) was constantly dropped under magnetic stirring (1200 rpm) to the chitosan-TPP nanoparticle solution (1 mL hormone: 3 mL chitosan-TPP nanoparticles complex) for 2 h. A dynamic light scattering leaser and Doppler electrophoresis (Zetasizer Nano ZS, Malvern Instruments Ltd., Worcestershire, UK) were used to identify physicochemical characteristics (the size, polydispersity (PdI), and zeta potential) of chitosan-TPP nanoparticles conjugated or not with GnRH or PGF_2α_.

### 2.2. Animals and Experimental Design

This study was conducted at the Experimental Farm of the Faculty of Veterinary Medicine, Cairo University, Egypt. All experimental protocols and procedures were performed according to the regulations of the Veterinary Animal Care Committee of the Faculty of Veterinary Medicine, Cairo University (Ethical approval number: Vet CU 8/03/2022/423). Fifteen cyclic, multiparous Baladi goats of age 4.5 ± 0.5 years and with average body weight and body condition scores of 35.5 ± 1.5 kg and 3.42 ± 0.57, respectively, were used in this study. All goats were healthy with no reproductive difficulties. They were managed as one flock under the same optimal management conditions at the Faculty of Veterinary Medicine’s research farm. The study was conducted during the natural breeding season. Goats received their nutritional requirements according to NRC recommendations [13].

During the experiment, goats were separated into three equal experimental groups (*n* = 5/group). The first group (GPG) received common Ovsynch protocol (an IM injection of 50 mg gonadorelin, followed by an IM injection of 125 mg cloprostenol 7 days later, and a further IM injection of 50 mg gonadorelin 2 days later), whereas the second and third groups received the Ovsynch protocol using nanofabricated hormones with the same dosage (NGPG) or half dosage (HNGPG) of the common protocol.

### 2.3. Ultrasonography Evaluation of Ovarian Structures and Hemodynamic Pattern

Ovarian structures and ovarian artery and luteal blood flow were evaluated using a pulsed-wave Doppler ultrasound scanner equipped with a transrectal 5–7.5 MHz linear-array transrectal transducer (EXAGO, Echo Control Medical, Angoulême, France) in color and spectral modes. The standard velocity and Doppler filter of the Doppler ultrasound were set at 25 cm/s and 100 Hz, respectively. The gate cursor was set to 0.5 mm in width. The angle of intonation was 46° [14,15]. During the experimental period, one operator was responsible for ultrasonography examination to avoid variations in collected data. Goats were subjected to intensive ultrasonography examinations after each Ovsynch protocol injection. The response to the first GnRH injection was assessed by scanning ovaries 5 days after the first GnRH injection. The response to the PGF_2α_ injection was assessed by scanning ovaries 2 days after PGF_2α_ injection. Furthermore, ovaries were scanned again at 0, 12, and 24 h after the second GnRH injection (presenting follicular phase) and on days 5, 10, and 15 of the synchronized estrous cycle (presenting luteal phase).

In each scanning session, we recorded the number and size category of each visible ovarian follicles (small follicles, ≥2 to ≤3 mm in diameter; medium follicles, >3 to <5 mm in diameter; and large follicles, ≥5 mm in diameter). The number and diameters of corpora lutea (CLs) were also recorded. The time of the disappearance of the dominant follicle at the follicular phase was used to estimate the time of ovulation onset by calculating the time interval between the first observation of the dominant follicle at 0 time and the midway point of the last observation of the dominant follicle (at 12 or 24 h).

For hemodynamic pattern evaluation, the blood flow indices of both ovarian and luteal arteries were determined using the spectral mode at the same time as ovarian structure evaluation. The electronic caliper of the ultrasound recorded several diameters of follicles and CLs. The vascularization of the ovarian follicle and CL was determined by the color flow mode with the presence of a pulsed-wave spectral graph showing Doppler parameters [16]. Blood flow indices of both ovarian and luteal arteries was determined using the spectral mode. Pulsatility index (PI), resistance index (RI), and peak systolic velocity (PSV) were used as blood flow index parameters [14].

### 2.4. Determination of Ovarian Steroids and Nitric Oxide

Blood samples were collected via jugular vein puncture corresponding to the times of ultrasound examination. Serum was harvested and stored at −20 °C for hormonal and NO analyses. P_4_ (EIA-1561) and E_2_ (EIA-2693) were analyzed using competitive enzyme-linked immunosorbent assay kits (DRG Diagnostics GmbH, Marburg, HE, Germany). The sensitivity of the method was 0.055 ng/mL for P_4_ and 9.9 pg/mL for E_2_. The intra- and inter-assay correlation coefficients were, respectively, 6.86% and 5.59% for P_4_ and 2.71% and 9.39% for E_2_. For NO assay, each serum sample was combined with an identical quantity of freshly organized Griess reagent and incubated for 10 min at room temperature, and absorbance was measured at 560 nm using a microtiter plate reader with the assay sensitivity of 0.225 mmol/L [17].

### 2.5. Statistical Analysis

The data collected after the initial GnRH and PGF_2α_ injections, including ovarian response, ovarian and luteal blood flow indicators, and hormonal profile, were analyzed using the generalized linear model procedure of SAS (version 9 edition. Cary, NC: SAS Inst, Inc; 2004). The used model was y_ij_ = µ + T_i_ + e_ij_in, where y_ij_ is the observed value of the dependent variable, µ is the overall mean, T_i_ is the fixed effect of the i^th^ treatment (i = 1:4), and e_ij_ is the residual error. Least-squares procedures using a mixed model, considering the time of data collection as repeated measurements, were used to assess the effect of estrous synchronization protocols on ovarian response, ovarian and luteal blood flow indices, and hormonal profile during the follicular and luteal phases of the synchronized estrous cycle. The treatment (T, estrous synchronization protocol: GPG, NGPG, and HNGPG), the day of blood sampling and/or ovarian examination (S), and their interaction (T × S) were introduced as fixed effects and individual goats as random effects. The used model was y_ijk_ = µ + T_i_ + S_j_ + (T × S)_ij_ + e_ijk_, where y_ijk_ is the observed value of the dependent variable determined from a sample taken from each animal, µ is the overall mean, T_i_ is the fixed effect of the i^th^ treatment (i = 1:3), S_j_ is the fixed effect of the j^th^ sampling/examination time (j = 1:3), (T × S)_ij_ is the interaction between treatment and day of the estrous cycle, and e_ijk_ is the residual error. All results were shown as means (±SEM), and differences between the means of different experimental groups were detected using Duncan’s new multiple range test with a *p*-value of less than 0.05.

## 3. Results

### 3.1. Physicochemical Characteristics of Ovsynch Hormone Nanoparticles

Table 1 shows physicochemical characteristics of chitosan-TPP nanoparticles and hormone-loaded chitosan-TPP nanoparticles. Results showed that chitosan-TPP nanoparticles and hormone-loaded chitosan-TPP nanoparticles were in nanosize (≈100 to 200 nm). The addition of hormones to the chitosan-TPP nanoparticles increased their size, indicating the binding of both molecules together. The range of PdI values of chitosan-TPP nanoparticles hormone loaded-chitosan-TPP nanoparticles was between 0.288 and 0.545. All fabricated nanoparticles (free or hormone-loaded) had positive zeta potential values.

### 3.2. Effect of Common or Nanodelivered Ovsynch Protocols on Ovarian Structures

In response to the first GnRH injection, the numbers of the small, medium, total follicles, and numbers of CLs were similar among the experimental groups, whereas the numbers of large follicles decreased (*p* = 0.024) in NGPG and HNGPG, compared with GPG. HNGPG had greater diameter of CLs (*p* = 0.001) than GPG and NGPG (Table 2).

After PGF_2α_ injection, the total number of follicles decreased (*p* = 0.038) in NGPG compared with GPG, whereas HNGPG recorded an intermediate value. The diameter of CLs was greater (*p* = 0.001) in HNGPG than in GPG and NGPG (Table 2).

During the follicular phase, the numbers of small and large follicles, numbers of total follicles, and diameter of dominant follicles were similar among the experimental groups (Table 2). HNGPG increased (*p* = 0.043) the number of medium follicles compared with GPG and NGPG. HNGPG shortened (*p* = 0.04) the time interval between the second GnRH and onset of ovulation compared with GPG and NGPG (Table 2). During the luteal phase, numbers of CLs were similar among different experimental groups, whereas the diameter of CLs increased (*p* < 0.001) in HNGPG, compared with GPG and NGPG (Table 2).

### 3.3. Effect of Common or Nanodelivered Ovsynch Protocols on Hemodynamic Indices

In response to the first GnRH injection, HNGPG decreased (*p* = 0.001) the ovarian artery pulsatility index (PI) and resistance index (RI), compared with GPG and NGPG. HNGPG decreased (*p* = 0.044) the luteal artery RI and increased (*p* = 0.041) PSV, in comparison with GPG and NGPG (Table 3).

In response to PGF_2α_ injection, HNGPG decreased ovarian artery PI (*p* = 0.003) and RI (*p* = 0.002), whereas increased (*p* = 0.003) luteal artery RI compared to GPG and NGPG (Table 3).

**Figure 1:**
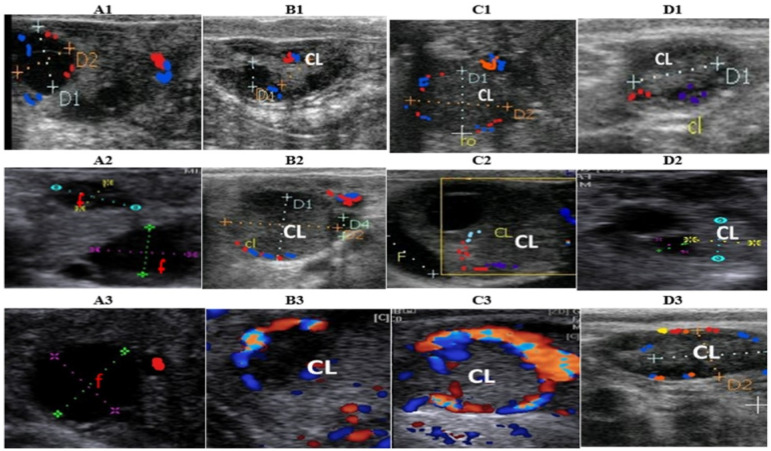
B-mode and colored ultrasonograms showing the preovulatory dominant follicle (Follicle: f; **A1**, **A2**, and **A3**) at the follicular phase and corpus luteum (CL) on days 5 (**B1**, **B2**, and **B3**), 10 (**C1**, **C2**, and **C3**), 15 (**D1**, **D2**, and **D3**) at the luteal phase in GPG, NGPG, and HNGPG protocols, respectively. Blue colored areas refer to the blood flow away from the probe (blood supply towards the organ/tissue) and red colored areas refer to the blood flow towards the probe (blood return from the organ/tissue towards blood circulation).

During the follicular phase, the least (*p* = 0.003) Doppler indices (PI and RI) of ovarian artery associated with greatest (*p* < 0.001) PSV were observed in HNGPG, whereas opposite patterns of Doppler indices were observed for the luteal artery (Table 3 and Figure 1). During the luteal phase, PSV of the ovarian artery declined (*p* < 0.001) and was associated with a significant elevation of both Doppler indices (*p* < 0.001) in HNGPG, whereas opposite patterns of Doppler indices were observed for the luteal artery (Table 3 and Figure 1).

### 3.4. Effect of Common or Nanodelivered Ovsynch Protocols on Hormonal Profile and Nitric Oxide Levels

In response to the first GnRH injection, both NGPG and HNGPG increased (*p* < 0.001 and *p* < 0.001; respectively) blood serum E_2_ and NO levels, compared with GPG. HNGPG increased (*p* < 0.001) levels of blood serum P_4_, compared with GPG, whereas NGPG resulted in an intermediate value (Table 4).

In response to PGF_2α_ injection, both NGPG and HNGPG increased (*p* = 0.007) NO levels, compared with GPG (Table 4). Blood serum E_2_ levels increased (*p* = 0.028) in HNGPG, compared with GPG, whereas NGPG resulted in an intermediate value (Table 4).

During the follicular phase, the least blood serum P_4_ level was observed in NGPG. HNGPG increased (*p* < 0.001) blood serum E_2_, P_4_, and NO levels, compared with GPG and NGPG. During the luteal phase, blood serum P_4_ and NO levels increased (*p* < 0.001 and *p* = 0.020; respectively) in HNGPG, compared with GPG and NGPG, whereas no changes in blood serum E_2_ levels were observed among the experimental groups (Table 4).

## 4. Discussion

In this study, we aimed to evaluate the efficiency of the Ovsynch estrous synchronization protocol as one of the most crucial estrous synchronization protocols in goats when GnRH and PGF_2α_ are delivered using a nano-based drug delivery system. Several studies have reported the advantages of engineered nanodrugs, including hormones, and their efficient biological activities due to the changes in physicochemical properties of materials in nanoforms [18,19]. Engineered nanohormones have a longer half-life time and sustained release, greater cellular uptake, and more efficient passage across epithelial or endothelial barriers [1]. These advantages can improve the delivery of the hormone to the target sites and thus the final action of the hormone. Most nanoparticles used in delivery systems are 50–250 nm in size. This size allows particles to move through various barriers and cell pores with ease, enhancing cellular uptake [20]. In this context, the success of any estrous synchronization protocol primarily depends on the pharmacokinetics and pharmacodynamics hormones. The Ovsynch estrous synchronization protocol depends on the use of GnRH and PGF_2α_. Given the fact that both hormones have a short lifespan and low molecular weight, they are susceptible to degradation easily by different lytic enzymes of the systemic circulation, restricting the sustained delivery of the hormones to the target sites and therefore their biological activity.

In this study, the conjugation of GnRH and PGF_2α_ to chitosan-TPP nanoparticles resulted in particle size of ≤200 nm, acceptable PdI (<0.6), and positively charged nanoparticles with acceptable zeta potential (>15 mV) for both hormones [9,21]. These properties can facilitate the delivery and uptake of both hormones by their target organs. For GnRH, where the brain is the main target organ, such physiochemical properties are suitable for efficient drug delivery to the brain. Nanoparticles with sizes ranging from 50 to 200 nm, a PdI ≤ 0.4, and a positive surface charge (up to 15 mV) are efficient for drug delivery to the brain [22]. These results can be confirmed in our study and several previous studies, as nano-GnRH even if used with a low dosage improved several reproductive events in farm animals, such as ovulation [12] and CL luteinization [9].

The main target organ for PGF_2α_ is the CL, and the main barrier to PGF_2α_ activity is its rapid degradation through the circulatory system, particularly the pulmonary system. However, in this study, the reduction of PGF_2α_ dosage to half did not adversely affect its luteolytic activity. To the best of our knowledge, there are no available studies in the field of estrous synchronization of farm animals that discussed the biological efficiency of nanoengineered PGF_2α_. However, some studies referred to the importance of transforming prostaglandin analogs to ensure the accumulation and sustained release of these hormones a long time [23,24].

Results of this study show the relevance of nanoengineered GnRH and PGF_2α_ in improving the response of the ovary, ovarian hemodynamic patterns, and hormonal profile to the Ovsynch protocol after each injection, resulting in better ovulatory wave characteristics and subsequent luteal functions of the synchronized cycle. In Ovsynch protocol, the supposed role of the first GnRH injection is to trigger ovulation of the LH-responsive follicle and/or to luteinize growing follicles. In this study, the first GnRH in both NGPG and HNGPG decreased the number of large follicles, compared with GPG. This is one of the intended effects of the first GnRH injection, as the termination of the existing follicular wave enables the development of a new follicular wave.

In this study, the first GnRH injection of HNGPG increased the diameter of CLs, as well as P_4_ levels on day five after the first GnRH injection. These effects may be related to the positive effects of the first GnRH of HNGPG on ovarian hemodynamic patterns (increased PSV and decreased PI and/or RI). The improvements in ovarian hemodynamic patterns can be ascribed to the increased levels of blood serum E_2_ and NO. Both E_2_ and NO have vasodilatation effects, resulting in improved vascular blood flow, supporting the development of ovarian structures and the hormone synthesis and secretion [14]. The level of P_4_ during the emergence of the new follicular wave is a crucial factor in the development of a new follicular wave, as greater P_4_ levels stimulate follicular turnover and boost the growth of a new follicular wave [25,26].

In this study, two main findings should be highlighted in response to the PGF_2α_ injection. First, the decrease in blood serum P_4_ levels was not associated with a decrease in the number and/or diameters of CLs 2 days after PGF_2α_ injection, compared with those recorded before the PGF_2α_ injection, in all experimental groups. These results are in agreement with those previously reported on the mechanism of PGF_2α_-induced luteolysis.

The luteolysis process is divided into two stages: a rapid drop in the functionality of the CL, resulting in lower P_4_ levels, and a phase of structural regression, resulting in luteal tissue shrinkage [27]. Second, both NGPG and HNGPG improved blood flow of the ovarian artery and increased blood serum E_2_ and NO levels, when compared with GPG. Classically, PGF_2α_ induces luteolysis by decreasing luteal blood flow, resulting in hypoxia; however, before the initial steps of the luteolytic cascade, PGF_2α_ increases luteal blood flow and CL vascularization [28,29]. Additionally, the increased blood serum E_2_ and NO levels in NGPG and HNGPG may contribute to this effect through their vasodilation effects [30,31]. These findings support the relevance of the PGF_2α_ in nanoform for the expected role of this injection even when the dose is reduced to half of the conventional dose. Therefore, it can be said that administering PGF_2α_ using a nanodelivery system may improve the biological function of PGF_2α_.

One of the parameters used for judging the effectiveness of the Ovsynch protocol is the ability of the protocol to induce tighter synchrony of ovulation and to shorten the time required from the second GnRH injection to ovulation. Tighter and shorter ovulation periods allow for easier applications of timed artificial insemination, as fertility variations due to scattering ovulations relative to insemination time are minimized, avoiding ova aging after ovulation [3,8]. In our study, HNGPG significantly reduced the time interval between the second GnRH and ovulation, resulting in one of the most crucial goals of the Ovsynch protocol. HNGPG-treated goats had better ovulatory wave characteristics, larger diameters of dominant and subdominant follicles, increased number of medium follicles, and higher blood serum E_2_ levels compared to GPG and NGPG. These effects can be associated with various positive effects of nanodelivered hormones on ovulatory follicle characteristics, ovarian blood flow, and the hormonal milieu during ovulatory wave emergence. HNGPG increased blood serum P_4_ levels during the emergence of the ovulatory wave, the period after the first GnRH injection. Elevated levels of blood serum P_4_ for the short term (5–7 days) are critical for stimulating ovarian follicles turnover and emergence of new ovulatory waves [25]. Additionally, these positive effects may be associated with increased levels of blood serum E_2_ and NO and the subsequent improvements in ovarian artery blood flow [32].

Interestingly, the positive effects of HNGPG extended to the luteal phase of the synchronized estrous cycle, as goats of this group showed the greatest diameter of CLs and blood serum P_4_ levels. One reason restricting the efficiency of an estrous synchronization protocol is the inadequacy of CL functioning and/or formation of short-lived CL, inhibiting subsequent fertility and pregnancy maintenance. In species like goats, the CL is the main source of P_4_, which is required during the entire pregnancy for the completion of a successful pregnancy [6]. The improved structure and functionality of CL during the luteal phase of the synchronized estrous cycle in HNGPG can be ascribed to the improved luteal blood supply, as the vascularization pattern of CL blood vessels and endothelial cells play a crucial role in CL function [33]. In this context, NO has been identified as a major mediator of increased CL blood flow in cattle [34]. Moreover, several studies have reported the ability of GnRH administered around ovulation time and/or during the early luteal phase to improve the functioning of newly formed CL by triggering the release of pituitary luteinizing hormone, LH, and subsequent luteinization of granulosa and theca cells [35]. This effect depends on the ability of GnRH to induce sufficient gonadotropin release [23], mainly LH, from the pituitary gland; consequently, long-lasting release of GnRH, as expected, following transforming GnRH to nanoform, may improve its biological efficiency by facilitating sustained surge of gonadotropins.

Finally, the question that should be addressed in this study is why NGPG did not improve the characteristics of the ovulatory follicle, CL structure and functionality, and HNGPG. In fact, there is no elucidation of these findings. However, it can be speculated that providing GnRH and/or PGF_2α_ by a nanodelivery system with the same doses used in conventional Ovsynch protocol could result in improper responses. Long-lasting release of the hormones may lead to receptors’ refractions and a lack of response to the hormone [36]. Nevertheless, to confirm or reject this assumption, more studies are required to investigate the dosage effect, release pattern of the nanofabricated hormones in the biological systems, and the interplay between these factors and animal response. Moreover, future studies have to show more information on the effect of such protocol on the reproductive performance of goats after insemination and subsequent fertility and pregnancy outcomes.

## 5. Conclusions

Conclusively, the results of this study confirmed the ability of HNGPG treatment to enhance the ovarian and luteal blood flow at the follicular and luteal phases, characteristics of the ovulatory wave, E_2_ synthesis at the follicular phase, and corpus luteum function and P_4_ synthesis at the luteal phase. Accordingly, the nanodelivery system for hormones of Ovsynch protocol can be recommended as a new promising reproduction management practice for improving Ovsynch protocol estrous synchronization outcomes of goats.

## Figures and Tables

**Table 1:** Physicochemical properties (particle size, polydispersity index (PdI), and zeta potential) of chitosan-TPP nanoparticles, gonadotropin-releasing hormone (GnRH)-chitosan-TPP nanoparticles, and prostaglandin F_2α_ (PGF_2α_)-chitosan-TPP nanoparticles.

Nanoparticles	Particle Size (nm)	PdI	Zeta Potential (mV)
Chitosan-TPP	110.25 ± 9.24	0.288 ± 0.05	17.36 ± 0.245
GnRH-chitosan-TPP	200.5 ± 4.25	0.442 ± 0.09	22.5 ± 0.24
PGF_2α_-chitosan-TPP	198.8 ± 15.20	0.545 ± 0.12	20.1 ± 0.31

**Table 2:** Ovarian response to Ovsynch protocol using conventional hormones (GPG: 50 mg gonadorelin and 125 mg cloprostenol) or nanodelivered hormones with different doses (NGPG: 50 mg gonadorelin and 125 mg cloprostenol and HNGPG: 25 mg gonadorelin and 62.5 mg cloprostenol) (means ± standard error of the mean, SEM).

Variable	Treatment (T)			SEM	*p*-Value
	GPG	NGPG	HNGPG		T
Response to the first GnRH injection					
Number of small follicles (≥2 to 3 mm)	6.00	5.80	6.40	0.181	0.585
Number of medium follicles (≥2 to ≤3 mm)	6.40	6.00	6.60	0.148	0.315
Number of large follicles (≥5 mm)	3.00 ^a^	2.00 ^b^	1.80 ^b^	0.256	0.024
Number of total follicles	15.40	13.80	14.80	0.535	0.293
Number of corpora lutea	2.00	2.00	2.60	0.145	0.092
Diameter of corpus luteum (mm)	4.51 ^b^	4.27 ^b^	5.84 ^a^	0.125	0.001
Response to PGF_2α_ injection					
Number of small follicles (≥2 to ≤3 mm)	5.80	5.60	5.40	0.106	0.493
Number of medium follicles (>3 to <5 mm)	6.00	5.20	5.80	0.151	0.198
Number of large follicles (≥5 mm)	3.40	3.00	3.20	0.117	0.564
Number of total follicles	15.20 ^a^	13.80 ^b^	14.40 ^ab^	0.191	0.038
Number of corpora lutea	2.00	2.00	2.60	0.117	0.148
Diameter of corpus luteum (mm)	4.48 ^b^	4.22 ^b^	5.78 ^a^	0.152	0.001
Follicular phase					
Number of small follicles (≥2 to ≤3 mm)	4.40	4.20	4.47	0.014	0.436
Number of medium follicles (>3 to <5 mm)	5.67 ^b^	5.33 ^b^	6.13^a^	0.203	0.043
Number of large follicles (≥5 mm)	3.20	3.47	3.33	0.186	0.607
Number of total follicles	11.92	11.56	12.39	0.303	0.174
Dominant follicle diameter (mm)	6.07	5.96	6.21	0.077	0.414
Diameter of secondary dominant follicle (mm)	5.38	5.42	5.82	0.098	0.106
Diameter of tertiary dominant follicle (mm)	5.08	5.12	5.42	0.081	0.105
Ovulation time (h)	15.60 ^a^	15.60 ^a^	10.82 ^b^	2.74	0.040
Luteal phase					
Number of corpora lutea	1.80	2.07	2.00	0.253	0.455
Diameter of corpus luteum (mm)	7.62 ^b^	7.81 ^ab^	8.03 ^a^	0.310	0.001

GnRH: gonadotropin-releasing hormone (gonadorelin), PGF_2α_: prostaglandin F_2α_ (cloprostenol), follicular phase: ovaries were scanned at 0, 12, and 24 h after the second GnRH injection, and luteal phase: ovaries were scanned on days 5, 10, and 15 of the synchronized estrous cycle. Within a row, means with different superscript letters (a and b) are significantly different (*p* < 0.05).

**Table 3:** Ovarian artery and luteal artery hemodynamic patterns in response to Ovsynch protocol using conventional hormones (GPG: 50 mg gonadorelin and 125 mg cloprostenol) or nanodelivered hormones with different doses (NGPG: 50 mg gonadorelin and 125 mg cloprostenol and HNGPG: 25 mg gonadorelin and 62.5 mg cloprostenol) (means ± standard error of the mean, SEM).

Variable	Treatment (T)			SEM	*p*-Value
	GPG	NGPG	HNGPG		T
Response to the first GnRH injection					
Ovarian artery					
PI	1.55 ^a^	1.53 ^a^	1.43 ^b^	0.013	0.001
RI	0.53 ^a^	0.53 ^a^	0.41 ^b^	0.014	0.001
PSV cm/s	16.03	14.48	15.01	0.203	0.134
Luteal artery					
PI	1.25	1.20	1.28	0.015	0.152
RI	0.57 ^a^	0.56 ^a^	0.44 ^b^	0.024	0.044
PSV cm/s	12.25 ^b^	12.27 ^b^	13.52 ^a^	0.187	0.041
Response to PGF_2α_ injection					
Ovarian artery					
PI	1.55 ^a^	1.54 ^a^	1.45 ^b^	0.012	0.003
RI	0.63 ^a^	0.62 ^a^	0.55 ^b^	0.014	0.002
PSV cm/s	12.94 ^b^	12.95 ^b^	14.65 ^a^	0.219	0.003
Luteal artery					
PI	1.25	1.23	1.28	0.013	0.364
RI	0.57 ^b^	0.57 ^b^	0.66 ^a^	0.014	0.047
PSV cm/s	12.75	12.42	13.08	0.214	0.438
Follicular phase					
Ovarian artery					
PI	0.91 ^a^	0.88 ^a^	0.81 ^b^	0.025	0.003
RI	0.85 ^a^	0.81 ^a^	0.71 ^b^	0.006	<0.001
PSV cm/s	9.76 ^b^	9.77 ^b^	10.62 ^a^	0.111	<0.001
Luteal artery					
PI	1.24 ^b^	1.25 ^b^	1.32 ^a^	0.024	0.020
RI	0.61 ^b^	0.64 ^a^	0.66 ^a^	0.022	0.019
PSV cm/s	12.52 ^a^	12.57 ^a^	12.01 ^b^	0.320	0.005
Luteal phase					
Ovarian artery					
PI	1.32 ^b^	1.38 ^b^	1.51 ^a^	0.025	<0.001
RI	0.59 ^b^	0.60 ^b^	0.64 ^a^	0.016	<0.001
PSV cm/s	12.25 ^a^	12.17 ^a^	11.25 ^b^	0.164	<0.001
Luteal artery					
PI	1.04 ^a^	1.02 ^b^	1.02 ^b^	0.017	<0.001
RI	0.48 ^a^	0.45 ^b^	0.41 ^c^	0.007	<0.001
PSV cm/s	13.59 ^b^	13.83 ^b^	14.53 ^a^	0.071	<0.001

GnRH: Gonadotropin-releasing hormone (gonadorelin), PI: pulsatility index, RI: resistance index, PSV: peak systolic velocity, PGF_2α_: prostaglandin F_2α_ (cloprostenol), follicular phase: ovaries were scanned at 0, 12, and 24 h after the second GnRH injection, and luteal phase: ovaries were scanned on days 5, 10, and 15 of the synchronized estrous cycle. Within a row, means with different superscript letters (a and b) are significantly different (*p* < 0.05).

**Table 4:** Blood serum levels of ovarian steroids and nitric oxide in response to Ovsynch protocol using conventional hormones (GPG: 50 mg gonadorelin and 125 mg cloprostenol) or nanodelivered hormones with different doses (NGPG: 50 mg gonadorelin and 125 mg cloprostenol and HNGPG: 25 mg gonadorelin and 62.5 mg cloprostenol) (means ± standard error of the mean, SEM).

Variable	Treatment (T)			SEM	*p*-Value
	GPG	NGPG	HNGPG		T
Response to first GnRH injection					
Progesterone (ng/mL)	5.11 ^b^	5.18 ^ab^	5.27 ^a^	0.397	<0.001
Estradiol (pg/mL)	24.11 ^b^	29.93 ^a^	31.08 ^a^	1.39	<0.001
Nitric oxide (μmol/L)	24.91 ^c^	31.25 ^b^	44.29 ^a^	3.52	<0.001
Response to PGF_2α_ injection					
Progesterone (ng/mL)	0.96	0.50	0.63	0.056	0.081
Estradiol (pg/mL)	31.07 ^b^	36.40 ^ab^	46.21 ^a^	1.41	0.028
Nitric oxide (μmol/L)	53.69 ^b^	62.90 ^a^	64.61 ^a^	1.38	0.007
Follicular phase					
Progesterone (ng/mL)	0.53 ^a^	0.45 ^b^	0.56 ^a^	0.100	<0.001
Estradiol (pg/mL)	36.64 ^b^	40.53 ^b^	46.66 ^a^	1.59	<0.001
Nitric oxide (μmol/L)	29.96 ^b^	32.42 ^b^	44.76 ^a^	0.475	<0.001
Luteal phase					
Progesterone (ng/mL)	4.36 ^b^	4.41 ^b^	4.92 ^a^	0.064	<0.001
Estradiol (pg/mL)	22.14	24.77	23.71	1.69	0.609
Nitric oxide (μmol/L)	35.38 ^b^	42.16 ^b^	54.27 ^a^	0.642	0.020

GnRH: gonadotropin-releasing hormone (gonadorelin), PGF_2α_: prostaglandin F_2α_ (cloprostenol), follicular phase: ovaries were scanned at 0, 12, and 24 h after the second GnRH injection, and luteal phase: ovaries were scanned on days 5, 10, and 15 of the synchronized estrous cycle. Within a row, means with different superscript letters (a, b, and c) are significantly different (*p* < 0.05).

## Data Availability

The data presented in this study are available on request from the corresponding author. The data are not publicly available because of privacy.
